# Gender Differences in the Pathogenesis and Outcome of Lupus and of Lupus Nephritis

**DOI:** 10.1155/2012/604892

**Published:** 2012-05-29

**Authors:** Julie Schwartzman-Morris, Chaim Putterman

**Affiliations:** ^1^Lupus and Arthritis Clinics, Division of Rheumatology, Jacobi Medical Center and North Central Bronx Medical Center, Bronx, NY 10461, USA; ^2^Division of Rheumatology, Albert Einstein College of Medicine, Bronx, NY 10461, USA

## Abstract

Systemic Lupus Erythematosus (SLE) typically affects females at far greater rates than males; however male SLE patients often have more severe disease than females. The gender disparities have been reported in clinical manifestations and in serological and hematological indices as well. In particular, SLE complicated with nephritis is more frequent in men than women, and several groups identified male gender as a risk factor for progression to renal failure. The specific differences in pathogenesis amongst genders have yet to be conclusively defined, though genetic, hormonal, and immune responses have been analyzed thus far. Further research is warranted to further elucidate these differences and permit the development of gender-tailored treatment regimens.

## 1. Introduction

Systemic Lupus Erythematosus (SLE) is a chronic autoimmune disease that can potentially cause inflammation and damage to any organ system. There are multiple genetic, hormonal, and environmental factors that are known to influence the development and nature of the disease. SLE is characterized by periods of high activity alternating with periods of remission and typically presents in females of childbearing age. During these reproductive years, the ratio of females to males is nine to one, with a lower ratio seen before puberty and a decline later in life. The increased rate of SLE in females implicates hormones as essential in disease manifestations, and this influence of sex hormones is also seen in animal models of the disease. Typically, in most mouse models, females have worse outcomes, and administration of estrogens exacerbates while androgens ameliorate disease [1]. 

## 2. Gender Differences in SLE Disease Manifestations

Despite the noted relationship of estrogens and increased autoimmune diseases in females, there is a growing body of the literature reflecting both different disease manifestations and a difference in severity of SLE in males versus females. Approximately 4% to 22% of SLE patients in reported series are male, and this number increases to 30% in studies regarding familial aggregation [2]. As a minority, males with SLE have been frequently subjected to treatments studied mostly in females, and become grouped along with females regarding most health-related issues. As gender differences may affect drug action and availability, tailored treatments for males and females might improve outcomes and overall prognosis for both genders [2]. Several groups have studied the sex disparities in this disease and have suggested gender, along with ethnicity, age of disease onset, or autoantibody profiles as a means to identify SLE subgroups [3]. More severe skin lesions, serositis, renal disease, thrombotic events, and seizures have been reported in males by several authors [4], though conflicting results have been presented regarding these gender differences among SLE patients and the precise role of gender in damage accrual has not yet been defined [4].

The LUpus in MInorities, NAture versus nurture (LUMINA) cohort is a well-known multiethnic US Cohort consisting of Hispanic, African American, and Caucasian patients. To further understand the impact of gender on manifestations and outcome of SLE, researchers in the LUMINA Study Group compared disease activity in males versus females by the SLAM (Systemic Lupus Activity Measure) and damage accrual by the Systemic Lupus International Collaborating Clinics/American College of Rheumatology Damage Index (SLICC/SDI) at baseline and at the last visit. In addition to the above disease indices, socioeconomic/demographic, clinical, and serological features were compared for this study [4]. 

Of the 618 LUMINA patients enrolled at the time of the study, 555 were female, 63 male. Caucasians were overrepresented amongst males. Poverty was less frequent, while smoking and alcohol use more common in males. Males in larger numbers experienced more difficulty in access to health care, but they showed more adequate illness-related behaviors at both points in time. These “illness-related behaviors” were measured using the Illness Behavior Questionnaire (IBQ), which assesses maladaptive responses to illness, including hypochondriacal responses, denial, and changes in affect. The IBQ was designed to indicate the extent to which these behaviors explain exaggerated response to health issues [5]. There was a tendency toward more frequent renal involvement (63.5% versus 52.1%, *P* = 0.085) and positive lupus anticoagulant antibody (LAC) in males (21.4% versus 9.1%, *P* = 0.004), but other antibodies occurred at comparable rates. Shorter disease duration at baseline, higher SDI at any time, and LAC at any time were factors independently associated with SLE in men (Table 1). Musculoskeletal involvement was less frequent in males in this cohort. Damage was shown to accrue faster in males with SLE; male sex was a stronger predictor of damage at baseline (55.6% versus 39.5%, *P* = 0.014) and positively associated with damage over the course of disease as measured at the last visit (71.4% versus 61.3%, *P* = 0.115). This study identified male gender as a risk factor for accelerated damage in SLE, in addition to reflecting differences in severity and manifestations of SLE by gender [4].

## 3. Gender Differences in Disease Manifestations: Focus on Renal Disease

Renal disease in SLE is a source of major morbidity and mortality; it develops in approximately 60% of patients with SLE, with a reported 5–22% of these patients progressing to end-stage renal disease requiring dialysis or transplant [6]. Studies have shown that lupus nephritis (LN) is more frequent in men than in women [7]. Multiple factors including male sex, black race, presence of antiphospholipid antibodies, increased creatinine at the time of diagnosis, anemia, frequent nephritic flares, hypertension, and excessive prolonged proteinuria are all considered risk factors for increased progression to end-stage renal disease (ESRD) [6]. However, the influence of gender on long-term renal outcome is controversial [7], and the differences in pathology of nephritis in males verses females have yet to be formally elucidated or studied. Higher prevalence, poorer renal outcome, and poorer overall survival rate have been demonstrated in several studies [8], as well as higher prevalence of Class IV, diffuse proliferative nephritis (DPGN), and active glomerular disease amongst males [2].

Lu et al. reviewed the existing literature published from 1975–January 2009 using the PubMed database to identify potential clinical characteristics in male lupus. Renal involvement was frequently more common in the studies reviewed, in both adult and pediatric SLE populations. In some, DPGN, the class with typically the poorest prognosis, was seen as the predominant finding on biopsy in males. Increased risk of renal failure and ESRD were also observed amongst males in greater numbers in various papers reviewed [2]. 

Of the patients in our own institution participating in the Einstein Lupus Cohort, approximately 300 SLE patients have been entered into the registry, of which 35 (11.7%) are males. Of the male patients, 16 (47%) have renal disease, whereas around 33% of the female patients have renal disease. Forty-one (46%) females have proliferative nephritis (class IV or mixed IV and V), 26 have class III or mixed III and V. Biopsy data on the remaining female patients is unavailable. Eight male patients (50%) have proliferative nephritis; seven have class IV, one with mixed class III and IV. Three males have mixed class III and V, two male patients class III. Two patients have pure membranous disease (class V) and one did not have a biopsy [9]. Our numbers are consistent with the other cohorts where males have renal disease in greater numbers and specifically more proliferative nephritis than the female counterparts.

## 4. Gender Differences by Geographic Region

### 4.1. Asia

 Several groups have analyzed the clinical expression and outcomes of SLE in males in different ethnic populations and geographic regions. To evaluate whether male patients in their local Chinese population had differences in clinical features at diagnosis, course of disease and features, rate and severity of disease relapses, organ damage, and cumulative damage scores, Mok et al. performed a retrospective review of 51 male patients and 201 female patients at the Rheumatology and Nephrology Clinics of the Queen Mary Hospital in Pokfulam, Hong Kong [1]. Disease activity was measured by the SLEDAI and organ damage by the SLICC/ACR Damage Index. At the time of diagnosis, there was a trend, but not a statistically significant difference, in the following: males had less arthritis, alopecia, anti-Ro antibody, less Raynaud's, and more discoid lesions and thrombocytopenia. Regarding renal disease, 11 males had renal biopsy at presentation; six male (55%) patients had DPGN, while 30 females had biopsies at presentation and 20 (67%) had DPGN, which was not a significant difference. There was also no difference in the presence of anti-double-stranded DNA antibodies on presentation. In this population, there was no difference in subsequent rate of development of DPGN; however, a significantly higher proportion of males had impaired renal function, with glomerular filtration rate (GFR) <50% normal, and a trend toward higher cardiovascular damage. The overall percentage of males requiring dialysis was not different from females. Total number of overall relapses was significantly less in males and severe flares also lower [1]. 

The above observation that disease course of SLE and male nephritis, aside from reduced GFR, was not different from males to females, differs from various other reports including another study of Acute Kidney Injury (AKI) in Chinese patients with lupus nephritis from a single center at the Peking University. AKI was defined as the presence of any one of the following items: an abrupt (within 48 hours) reduction in kidney function currently defined as an absolute change in serum creatinine of more than or equal to 0.3 mg/dL; a percentage increase in serum creatinine of ≥50% (1.5-fold from baseline); or a reduction in urine output (documented oliguria of less than 0.5 mL/kg per hour for more than 6 hours). AKI was previously identified as a risk factor for progression to ESRD. However, little was known about the patient characteristics of these patients or that gender may be a risk factor for AKI. The clinical, laboratory, renal histopathology, treatment, and outcome data were retrospectively collected and compared between lupus nephritis patients with and without AKI. The impact of AKI on renal outcome was evaluated [10]. 

Among 322 patients with renal-biopsy-proven lupus nephritis, 66 (20.5%) were identified as AKI. Male predominance was observed in patients with AKI (*P* < 0.001). The mean value of serum creatinine was 3.82 ± 2.59 mg/dL upon diagnosis. Most patients had hematuria (90.9%) and leukocyturia (71.2%). More than half of the patients presented with nephrotic syndrome (68.2%), with the amount of urine protein between 0.76 and 21.04 g/24 h (mean 6.57 ± 4.36). Regarding the pathological classification of lupus nephritis, the proportion of class IV was significantly higher (*P* < 0.001), and the proportion of classes III and V was significantly lower in the AKI group (*P* < 0.001 for both). In the AKI group, there was a significantly higher score of the total activity indices, endocapillary hypercellularity, cellular crescents, karyorrhexis/fibrinoid necrosis, subendothelial hyaline deposits, interstitial inflammation, leukocyte infiltration, the total chronicity indices, tubular atrophy, and interstitial fibrosis. In comparison with the non-AKI group, patients with AKI had significantly higher proportions of serositis (*P* < 0.001), neurologic disorder (*P* = 0.026), anemia (*P* < 0.001), thrombocytopenia (*P* = 0.013), and nephrotic syndrome (*P* = 0.011), but significant lower serum C3 (*P* < 0.001). The SLEDAI scores, renal pathological activity indices and, chronicity indices were significantly higher in the AKI group (*P* < 0.001 in all cases). 

Regarding outcome, the AKI group had a significantly poorer renal outcome compared with non-AKI group (*P* < 0.001). In the AKI group, patients with crescentic glomerulonephritis and thrombotic microangiopathy had the worst renal outcome. AKI was an independent risk factor for renal outcome along with male gender, age, activity index score, presence of crescents, chronicity score, interstitial inflammation, glomerular sclerosis, fibrous crescents, tubular atrophy, and interstitial fibrosis. The findings were consistent with the previous studies where mostly male lupus patients presented with more severe renal involvement and poorer outcome than female lupus patients, in clinical manifestations, laboratory characteristics, pathological features, and outcome of patients with AKI [10].

### 4.2. South America

Moving to a different geographic region and ethnic group, a case-control study assessing renal outcome of lupus nephritis in male patients at the Sao Paulo University Medical School in Brazil, by Resende, et al. was published in 2011 [11]. The primary endpoint was doubling of serum creatinine and/or end-stage renal disease. The secondary endpoint was defined as a variation of GFR per year, calculated as the difference between final and initial estimated GFR (eGFR) adjusted by follow-up time for each patient. At baseline, male and female patients were not statistically different regarding WHO LN class, eGFR, follow-up time, and 24-hour proteinuria, as well as age, albumin, C3, antinuclear antibody, anti-DNA antibody, and hematuria. There was no difference in the primary outcome, but male gender was significantly associated with a worse renal function progression, as measured by GFR per year calculated as the difference between final and initial estimated GFR (eGFR) adjusted by follow-up time for each patient. The multivariate linear regression model showed that male gender remained statistically associated with a worse renal outcome even after adjustment for eGFR, proteinuria, albumin, and C3 complement at baseline. 

The Grupo Latinoamericano de Estudio del Lupus (GLADEL) started in 1997 as a multinational inception prospective cohort in Latin American centers having expertise in the diagnosis and management of SLE. The data from the first 1214 patients was incorporated in a computer database available to all groups and interconnected among them. M. A. Garcia et al. used the data to analyze the influence of gender in the disease pattern and prognosis in a prospective cohort of SLE patients from 34 centers in nine Latin American countries: Argentina, Brazil, Colombia, Cuba, Chile, Guatemala, México, Peru, and Venezuela [12]. Of the 1214 SLE patients included in the GLADEL cohort, 123 were male. Demographic characteristics as well as clinical manifestations, laboratory profile, activity, and damage scores were evaluated at onset and during the course of the disease and compared with female patients. The median age at onset of the male patients was 27 and that at diagnosis 29.2 years. Delay to diagnosis was shorter in males (134 versus 185 days, *P* = 0.01). At onset, men more frequently showed constitutional symptoms and a higher prevalence of neurologic manifestations (4.5 versus 0.8%, *P* ≤ 0.053). During disease course, renal disease, characterized by persistent proteinuria and/or cellular casts, was significantly higher in males (58.5% versus 44.6%, *P* = 0.004), as was hemolytic anemia. Males more frequently had leukopenia, lymphopenia, thrombocytopenia, hemolytic anemia, IgG anticardiolipin antibodies, and low C3, along with any form of cardiovascular manifestation (56.1 versus 41.4%, *P* = 0.002), particularly arterial hypertension. Although not statistically significant, mortality was also higher in men [12]. Arthralgia and/or arthritis were more frequent among women (Tables 1 and 2). 

Molina et al. had found similar results in an earlier cross-sectional study of 107 Latin American male patients compared to a group of 1,209 Latin American female patients with SLE [8]. In this population at hospitals in Columbia and Mexico, the three most common findings in males were arthritis, skin, involvement, and renal disease. Renal involvement (58% versus 44%, *P* = 0.004) and vascular thrombosis occurred at significantly higher rates in males. The rate of nephrotic syndrome (31% versus 22%, *P* = 0.04) and the presence of dsDNA antibodies were significantly higher in males, as was the use of moderate-to-high doses of corticosteroids. Diffuse proliferative nephritis was the most common biopsy finding in both groups. Although there was no difference in mortality from all causes, SLE-related mortality was higher in the male group. The use of cytotoxic agents, dialysis, and renal transplantation was higher in the male group, though not statistically significant. Extrarenal manifestations more prevalent amongst males included CNS involvement, osteonecrosis, and severe cardiopulmonary disease.

### 4.3. Europe

Gender differences in SLE and nephritis were studied by two separate groups in Greece. The more recent retrospective study by Stefanidou et al. sought to analyze the prevalence of the most relevant clinical features of SLE in a sample of male patients as well as the incidence of the main causes of morbidity in a 5-year period after the diagnosis. Another goal was to investigate the impact of gender on expression and morbidity of SLE. Data were collected from the medical records of 59 male and 535 female patients with SLE who were diagnosed at the hospitals in the region of Thessaloniki. Several differences in the expression and morbidity of the disease were found in relation to the gender of the patient. Male patients had a higher prevalence of thromboses, nephropathy, strokes, gastrointestinal tract symptoms, and antiphospholipid syndrome when compared with female patients, but tended to present less often with arthralgia, hair loss, Raynaud's phenomenon, and photosensitivity as the initial clinical manifestations. The rates of nephropathy were 27.1% in males versus 16.1% in females (*P* = 0.002, OR = 2.806, 95% CI = 1.462–5.382). Specific details about class of nephritis or progression to ESRD and dialysis were not provided. The rates of thrombosis were 20.3% versus 4.7% (*P* < 0.001, OR = 5.832, 95% CI = 2.698–12.608), and stroke 8.5% versus 0.9% (*P* < 0.001, OR = 12.289, 95% CI = 3.176–47.55). During the 5-year followup, positive associations were noted between male gender and the incidence of tendonitis, myositis, nephropathy, and infections, particularly of the respiratory tract [13].

Voulgari et al. published a study of 489 Greek patients who visited the Rheumatology Department of the University Hospital of Ioannina, Greece, between 1981 and 2000. Four hundred and twenty-one were female (86%), while 68 were male (14%). There were no significant differences in the mean age at presentation, mean age at disease onset, duration of the disease, or in duration of followup between men and women. At the time of diagnosis, there were no major differences in major organ involvement; during followup, men had a greater frequency of renal involvement in younger patients. Eight men (14%) and 25 women (7%) developed chronic renal failure, while 3 men (5%) and women (1%) developed end-stage renal disease. No other major organ differences were observed between genders [14].

### 4.4. North America

The study of SLE within the United States Military presents a unique opportunity for examining clinical variables in the period before diagnosis. In addition, the demographics of the United States Military increase the expected proportion of male SLE patients. All military personnel have readily accessible health care and are required to receive regular physical exams, minimizing the impact of health care disparities and, to a lesser extent, of socioeconomic and cultural differences. The US Department of Defense serum repository contains samples from active duty personnel at entry into the military and, on average, every other year thereafter. Arbuckle et al. used this data to make comparisons between demographic, serologic, and clinical manifestations, as well as to identify factors associated with the manifestations of disease during the clinical period after the initial presentation to diagnosis of lupus. A cohort was assembled of 130 individuals diagnosed with SLE while on active duty in one of the United States uniformed services, with the goal of identifying potential subsets within the patient population [3]. 

Sixty-five of the patients were women; among the entire population 62% were African American (AA) and 26% were European American (EA). Asians and Hispanics comprised three and 9%. Individual gender subgroups contained sufficient numbers to permit separate analyses for AA and EA men and women. No difference in the time of the occurrence of the first criterion to diagnosis was based upon ethnicity alone; however, the time from this first manifestation to diagnosis was significantly shorter for males when compared with females. Evaluation of AA males in whom the diagnosis was made 0.17 years after the presence of their first clinical criteria suggests that this difference was due almost exclusively to AA males. Eighty-seven percent of AA males met SLE diagnostic criteria less than six months after their initial presentation criteria compared with 42% of AA females (*P* = 0.001; OR = 9.2). Manifestations at presentation and during followup were identified for AA males and compared to others (AA females and EAs) in order to assess for clinical variables that might contribute to the rapid onset observed in AA males. AA males had more anti-RNP antibodies, developed more nephritis and had less cutaneous manifestations than AA females and EAs. African American males were much more likely to present with nephritis, pleuritis, and/or seizures than others [3].

In another US study, Crosslin and Wiginton utilized hospital discharge data collected during a seven-year period to determine the effect of gender on SLE comorbidities and disease severity. Patients were hospitalized in the Dallas-Fort Worth metropolitan area between 1999 and 2005 and had a diagnosis of SLE. The sample consisted of 14,829 patients with SLE, 10% of which were male. Differences between males and females for disease severity, age, length of stay in the hospital, total hospital charges, and number of autoimmune diseases were studied. Disease severity was measured with the SLE comorbidity index, which weights 14 conditions in SLE. Male patients had significantly greater disease severity compared with female patients, while female patients had more autoimmune diagnoses compared with male patients. Male patients were more likely to have cardiovascular and renal comorbidities compared with female patients. Female patients had significantly greater odds of diagnoses of urinary tract infection, hypothyroidism, depression, esophageal reflux, asthma, and fibromyalgia. Males had greater odds of acute and chronic renal failure and nephritis, specifically, as well as thrombocytopenia, CHF, and arrhythmias. These findings corroborated those of previous studies of male severity of renal involvement in SLE [15]. 

Most recently, Tan et al. published a study of comparing male and female patients with SLE in the Hopkins Lupus Cohort. The cohort consisted of 157 men (66.2% white, 33.8% African American) and 1822 women (59.8% white, 40.2% African American).The mean followup was 6.02 years (range 0–23.73). Men were more likely than women to have hypertension, thrombosis, renal, hematological, and serological manifestations. Specifically increased in males were lymphopenia, positive anti-Sm, direct Coombs, lupus anti-coagulant, low C3, and anti-dsDNA. Men experienced increased rates of end-organ damage including neuropsychiatric, renal, cardiovascular, peripheral vascular disease, and myocardial infarction, and to have died. Women were more likely to have malar rash, photosensitivity, oral ulcers, alopecia, Raynaud's phenomenon, or arthralgia [16].

## 5. Gender Differences in Disease Manifestations: Pediatric Lupus

In the pediatric SLE population, as previously stated, the gender difference in numbers of patients is much reduced, to a ratio of three to one females to males before puberty. Several studies have reported that children have often an aggressive clinical course with more frequent renal involvement as compared to adults. Boys were reported to have a higher prevalence of severe renal disease and poorer outcome, but other reports did not confirm these findings [17]. Al-Mayouf and Sonbul sought to determine the influence of gender and age of onset on the outcome in Saudi children with SLE [17]. Outcome measures included SLICC/ACR, renal disease requiring dialysis, or transplant and death related to SLE. Patients were classified based on age at disease onset into early onset (<5 years) and late onset (>5 years). Eighty-nine patients (76 female and 13 male) were included, with median disease duration of 5 years. Twelve patients had early-onset disease. There was no difference in the mean age, age at diagnosis, disease duration, and followup between the different groups. Logistic regression analysis showed significant association of high SLICC/ACR score with early-onset disease and male gender, while renal disease requiring dialysis and renal transplant was associated significantly with male gender independently of age of disease onset. Death related to SLE was influenced by early-onset disease. Male children and early-onset disease of this cohort had poorer outcome [17].

## 6. Molecular Mechanisms That May Contribute to Gender Bias in Lupus: Estrogen and Its Receptors

As above, sex hormones are probably partly responsible for the higher occurrence of autoimmune disorders given the female predominance in autoimmune diseases. However, studies have found that sex hormone levels in patients with autoimmune disorders are not significantly different from patients without autoimmune disorders, indicating that other gender-associated differences including hormone regulation and effects on cytokine production, along with chromosomal factors, contribute to the high female predominance in these diseases compared with men [18]. Thus, the relationship of sex hormones increasing serum levels of certain cytokines and the estrogen receptor may be important in disease development [19]. 

Estrogen's primary effects are mediated via estrogen receptors alpha and beta (ER *α*/*β*) that are expressed on most immune cells. ERs are nuclear hormone receptors that can either directly bind to estrogen response elements in gene promoters or serve as cofactors with other transcription factors. ERs have prominent effects on immune function in both the innate and adaptive immune responses. Genetic deficiency of ER*α* in murine models of lupus results in significantly decreased disease and prolonged survival, while ER*β* deficiency has minimal to no effect in autoimmune models [20]. These two isoforms of ER are able to modulate the cytokine production of various key target cells of the immune system. 

ER*α* is expressed in most immune cells both at baseline and at increased levels after estrogen is given, in particular on antigen presenting cells. It can be detected in thymocytes, bone marrow nonhematopoietic cells, T cells, B-cell precursors, and circulating B cells, as well as dendritic cells (DCs) [20]. Estradiol can modulate lymphocyte cytokine production, cytokine receptor expression, and activation of effector cells. Estrogens favor the Th2 immune response, and enhanced interferon-*γ* (INF*γ*), TNF*α*, TGF*β*, interleukin (IL)-1, IL-5, IL-4, and IL-10 production. Estrogen causes a proliferation of M2 macrophages, and myeloid-derived suppressor cells, further amplifying the Th2 immune response [18]. Estrogen and prolactin are both capable of stimulating autoreactive B cells, promoting the failure of immune tolerance and secretion of autoantibodies [19]. 

Regarding DCs, Oertelt-Prigione found that exposure of immature murine DCs to estrogen increased their IL-6, IL-8, and MCP-1 production, but most importantly enhanced their stimulatory capacity on T lymphocytes, and another group demonstrated estrogen's role in enhancing the differentiation of DCs from bone marrow in vivo. Estrogen-driven upregulation of MHC II in the dendritic cells, enhancement of proinflammatory cytokine production, and an interaction between estrogen and MHCII expression regulation have also been confirmed in animal models [21]. 

Further evidence of sex hormones influence on systemic autoimmunity is derived from lupus animal models (NZB X NZWF1). In this model females develop disease earlier than males and die at younger age. Oophorectomy delays the disease onset while castrated males suffer of early and more severe disease; treatment with estrogens and prolactin causes early mortality. In comparison, mice treated with the estrogen receptor antagonist tamoxifen have a mild disease and a longer life [19]. In a recent murine study ER*α* KO genotype was bred onto three different murine lupus-prone strains, NZB/NZW f1, MRL/lpr, and NZM2410. In each of these three strains, the lack of ER*α* significantly attenuated disease. In NZB/NZW mice, the lack of ER*α* resulted in decreased autoantibody levels, while in NZM2410 and MRL/lpr mice, autoantibodies, if anything, were increased; the primary impact of ER*α* deficiency appeared to be on the response of the kidney to immune injury. When used in murine models of lupus, ER inhibitors were reported to have beneficial effects on lupus disease expression [20]. 

Additional details about estrogen-induced modulation of cytokine production in SLE mediated by the estrogen receptor and of the various aspects of estrogen receptor signaling in this disease, estrogen receptor subtypes, their structure, and the mode of action of estrogens by gene activation and via extranuclear effects are outlined in a recent review by Kassi and Moutsatsou [22].

## 7. Gender Differences in Pathogenesis

### 7.1. Genetics

As SLE affects more females than males, it is unexpected that the disease in males would be more severe. Given this predominance of the disease in females and its association with higher estrogen in both males and females, Hughes et al. sought to clarify why affected men often experience more severe disease by examining sex-specific genetic effects among SLE susceptibility loci. The group of male patients in this study was noted to have twice the risk of renal disease (OR = 1.70 (95% CI = 1.34 to 2.17, *P* = 1.2 × 10^−5^)) and more likely to have thrombocytopenia (OR = 2.26 (95% CI = 1.62 to 3.15, *P* = 5.7 × 10^−7^)) [23]. They investigated differences in allelic frequency between men and women using 18 previously identified independent autosomal genetic susceptibility loci for SLE. Genotyping was performed on over four thousand patients with SLE and nearly the same number of healthy controls. Sex-specific genetic association analyses and cumulative genetic risk scores for SLE in each individual were calculated to examine aggregate differences in sex-specific genetic risk. The genetic risk for SLE was significantly higher in males than females; more specifically, the frequency of two risk alleles in the HLA locus was significantly higher in males (rs3131379: OR male-female 1.37 (95% CI = 1.14 to 1.66),  *P* = 0.0010; rs1270942: OR male-female 1.40 (95% CI = 1.16 to 1.69), *P* = 0.00046). This was also the case for an SNP in IRF5 (rs2070197: OR male-female 1.23 (95% CI = 1.01 to 1.49), *P* = 0.039). There was no difference in the risk allele frequencies in the control group between men and women (*P* = 0.39, 0.52 and 0.64, for rs3131379, rs1270942, and rs2070197, resp.). 

### 7.2. Hormones

In many clinical and experimental scenarios, the incidence and the rate of progression of non-lupus-related renal diseases are influenced by multiple gender-dependent factors, such as kidney and glomerular size, differences in glomerular hemodynamics, and direct effects of sex hormones on renal tissue and signal pathways such as the renin-angiotensin-aldosterone system and signal molecules (e.g., nitric oxide, reactive oxygen species, cytokines, and growth factors) [24]. It has been shown that the main female hormone, 17*β* estradiol, is capable of inhibiting inflammatory and proapoptotic processes and protecting the renal tissue. In contrast, the male hormones, testosterone and dehydroepiandrosterone, have the opposite effect. Hormonal manipulation by male or female castration changes the course of renal disease progression and confirms the influence of the sex hormones. Female gender is therefore considered a protective factor in many kidney diseases, such as primary glomerulonephritis, autosomal dominant polycystic kidney disease (ADPKD), and hypertensive nephropathy [24]. 

Studies in patients with chronic kidney diseases have also shown that men have a more rapid disease progression and that with age, men exhibit greater decrements in renal function and increased glomerular sclerosis than women [25]. Women with several nondiabetic renal diseases such as membranous nephropathy, IgA nephropathy, and polycystic kidney disease present with a slower progression [25]. Thus, men appear to be at greater risk for renal injury than are women, though the exact reasons have not yet been established. It has been suggested that sex hormones mediate the effects of gender on chronic renal disease, through the interaction with the renin-angiotensin system, the modulation of nitric oxide synthesis, and the down regulation of collagen degradation. Androgens may contribute to continuous loss of kidney cells though the stimulation of programmed cell death which is activated in several chronic kidney diseases. Studies in vitro indicate that androgens prime a Fas/FasL-dependent apoptotic pathway in kidney tubule cells. The mechanisms to cell death which are primed by androgens may interact with others occurring in several conditions leading to the loss of renal cells. These findings are consistent with a role for androgens to promote chronic renal injury in men [25]; however, none of these findings have been directly connected to SLE renal disease.

Although the gender effect of dimorphism in lupus nephritis development has been often attributed to sex hormones as above, XXY males have nearly a 14-fold higher risk of developing SLE than 46 XY males, indicating that X-linked genes may also be risk factors for SLE in humans [26].

### 7.3. Toll-Like Receptors

Located at Xp22.2, Toll-like receptor 7 and its functionally related gene *TLR8* encode proteins that play critical roles in pathogen recognition and activation of innate immunity; they recognize endogenous RNA-containing autoantigens and when stimulated promote the expression of type I IFN, a pivotal cytokine in the pathogenesis of SLE. Animal models have demonstrated a connection between X-linked gene overexpression and TLR7. The BXSB strain of mice spontaneously develops an autoimmune syndrome with features of SLE; males are affected much earlier than females. Genetic analysis of the F1 hybrids of male BXSB mice with other lupus-prone mice demonstrated that the accelerated development of SLE in male BXSB mice is linked to the Y chromosome of the BXSB strain. This genetic abnormality present in BXSB Y chromosome has thus been called *Yaa*, Y-linked autoimmune acceleration [27]. 

The Yaa mutation was shown to be a consequence of a translocation from the telomeric end of the X chromosome and onto the Y chromosome [27]. Based on the presence of the gene encoding TLR7 in this translocated segment of the X chromosome, the possible role of TLR7 in the activation of autoreactive B cells and the development of SLE, the TLR7 gene duplication has been proposed to be the etiological basis for the Yaa-mediated enhancement of disease. Studies of Yaa and non-Yaa double bone marrow chimeric mice have demonstrated that anti-DNA autoantibodies are selectively produced by B cells bearing the Yaa mutation, and that T cells from both Yaa and non-Yaa origin efficiently promote anti-DNA autoantibody responses [27]. 

Amano et al. additionally demonstrated that the Yaa mutation causes defective development of marginal zone B cells in BXSB mice, suggesting a role for the marginal zone B cells in the generation of pathogenic autoantibodies in SLE [28]. Shen et al. have identified the association of a TLR7 SNP with SLE in 9274 Eastern Asians with a stronger effect in males than female subjects (odds ratio, male versus female = 2.33 (95% CI = 1.64–3.30) versus 1.24 (95% CI = 1.14–1.34); *P* = 4.1 × 10^−4^). Their data established a functional polymorphism in type I IFN pathway gene TLR7 predisposing to SLE, especially in Chinese and Japanese human male subjects [26].

## 8. Summary and Conclusions

The impact of gender in SLE renal disease has been assessed thus far, mostly with chart reviews and retrospective analyses. In order to further identify and clarify the true and significant differences in pathogenesis, prognosis, and long-term outcome, more systematic and prospective studies should be undertaken. Both nonrenal (Table 1) and renal manifestations (Table 2) have been compared in male versus female patients in several cohorts. It seems clear that both in the adult and pediatric lupus populations, male patients have greater disease severity, including rapid clinical progression to diagnosis, progression to renal injury and failure, and greater renal-related morbidity. Separation by gender for future studies of treatment outcomes might serve to identify which of the many existing and competing treatment strategies have the greatest benefit for each group, and to further identify which subgroups should be targeted for aggressive treatment at diagnosis.

## Figures and Tables

**Table 1:** Nonrenal manifestations of SLE more prominent in males by cohort.

Cohort	Laboratory abnormalities found increased in males	Clinical manifestations increased in males	Clinical manifestations decreased in males
LUMINA [4]	Lupus anticoagulant	Organ damage accrual	Musculoskeletal (MSK) disease

Hopkins [16]	Lymphopenia	Neuropsychiatric	Malar rash
	anti-Sm	Renal	Photosensitivity
	direct Coombs	Cardiovascular disease	Oral ulcers
	Lupus anti-coagulant	Peripheral vascular disease	Alopecia
	low C3		Raynaud's phenomenon
	anti-dsDNA		Arthralgia

Mok [1]	Thrombocytopenia	Discoid lesions	MSK disease
		Cardiovascular damage	Alopecia
			Raynaud's disease

GLADEL [12]	Leukopenia	Constitutional symptoms	Arthralgia
	Lymphopenia	Neurologic manifestations	MSK disease
	Hemolytic anemia	at onset	Skin disease
	Thrombocytopenia	Cardiovascular disease	
	IgG ACL Ab	Arterial HTN	
	Low C3		

Molina [8]	dsDNA ab	Arthritis	Raynaud's disease
		Vascular thrombosis	
		CNS manifestations	
		Cardiopulmonary disease	

Stefanidou [13]	Thrombocytopenia	Stroke	Alopecia
		APLS	Arthralgia
		GI symptoms	Photosensitivity
		Vascular thrombosis	Raynaud's disease

**Table 2:** Comparison of gender differences in four separate cohorts of SLE patients.

Cohort		Male	Female	*P* value
LUMINA [4]	Patient no. (total)	63	555	
	Renal involvement	63.5%	52.1%	*P* = 0.085

GLADEL [12]	Patient no. (total)	123	1091	
	Renal involvement	58.5%	44.6%	*P* = 0.004

Molina [8]	Patient no. (total)	107	1209	
	Renal involvement	58%	44%	*P* = 0.004

Stefanidou [13]	Patient no. (total)	59	535	
	Renal involvement	27.1%	16.1%	*P* = 0.002
